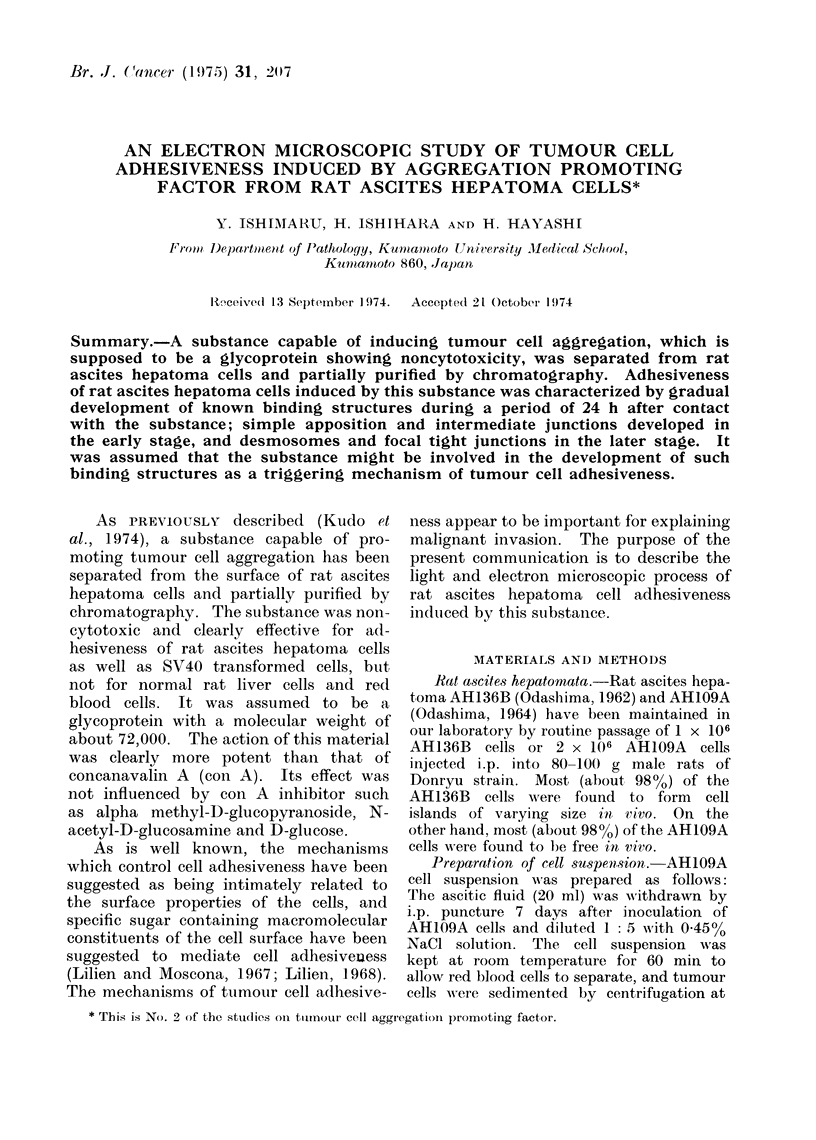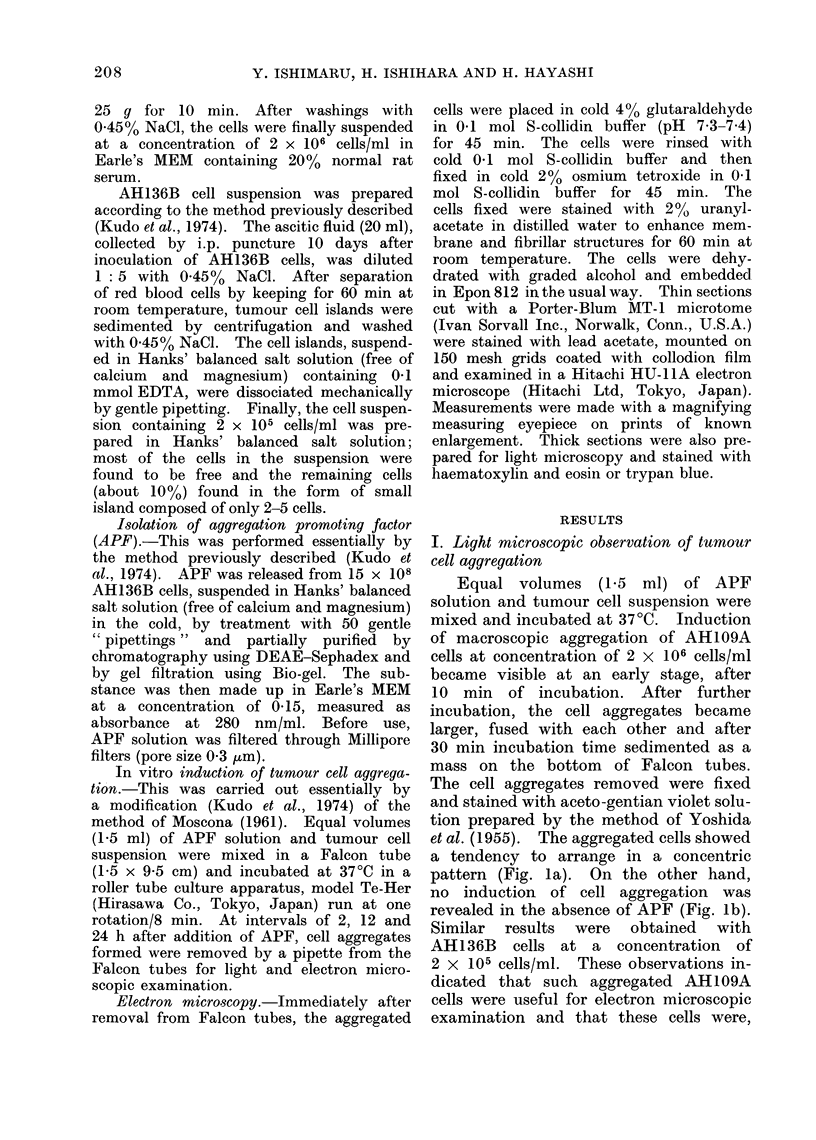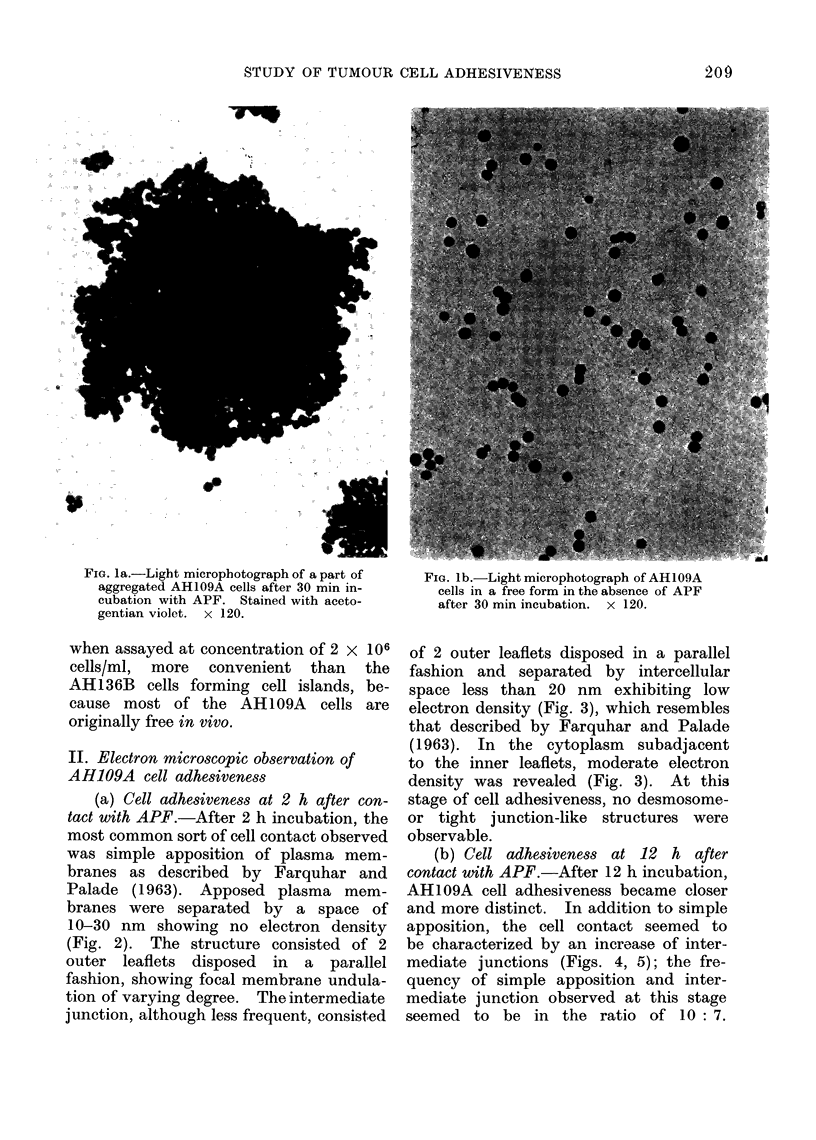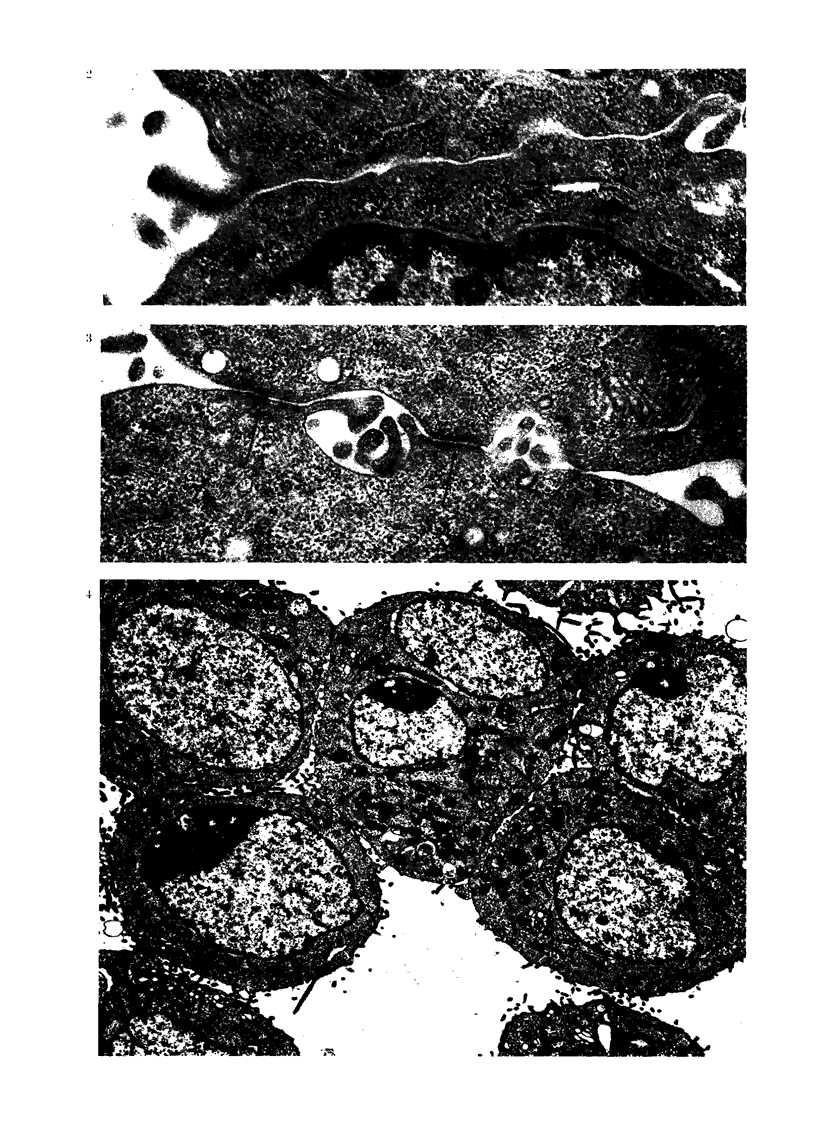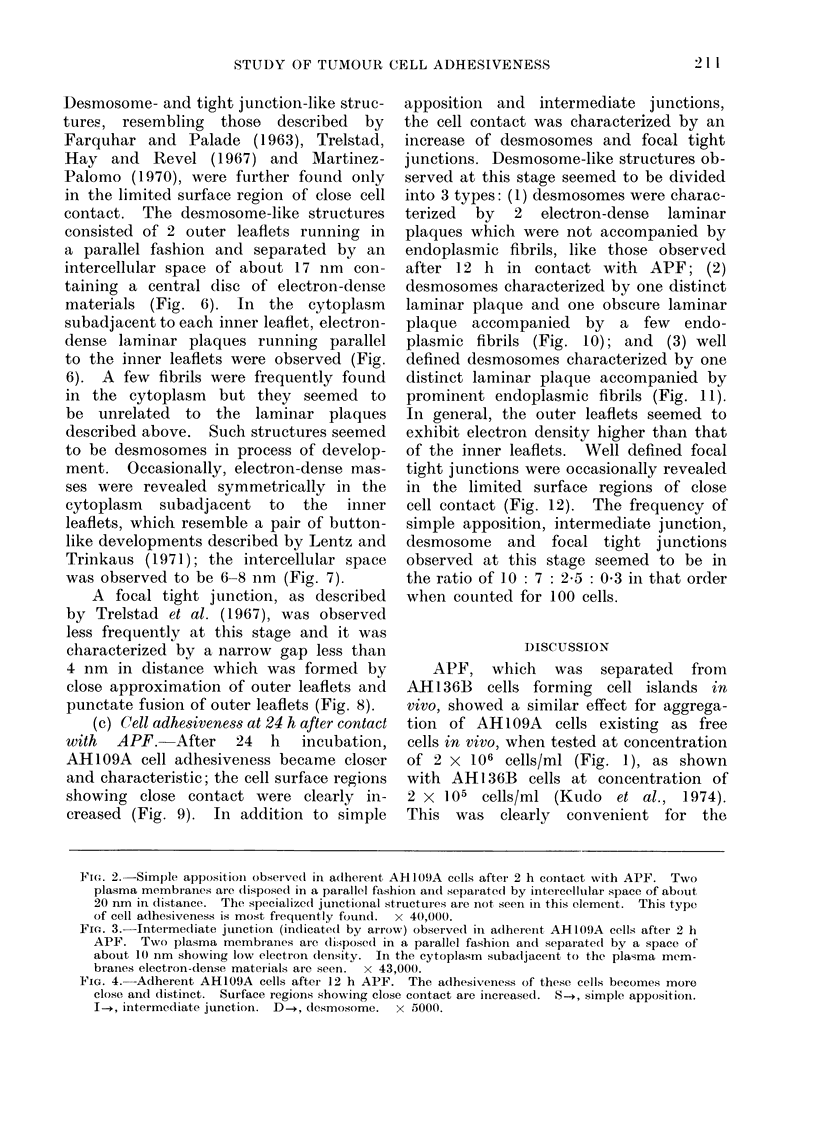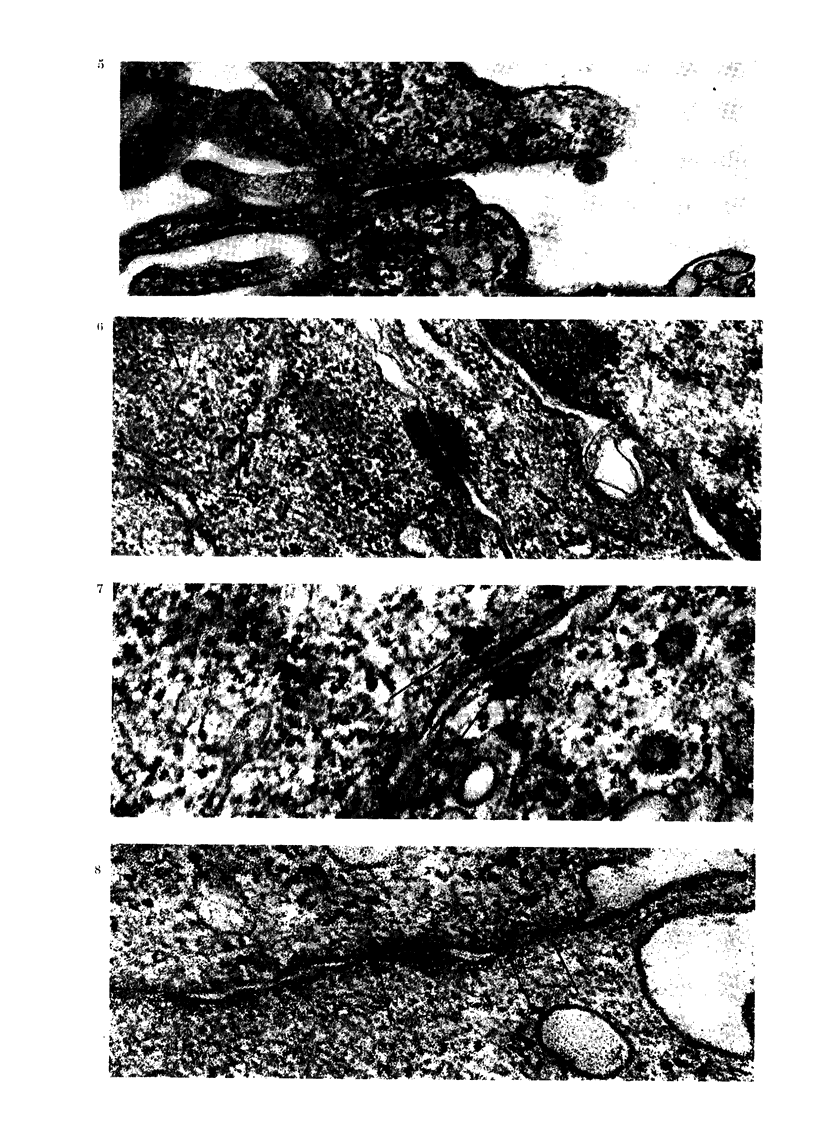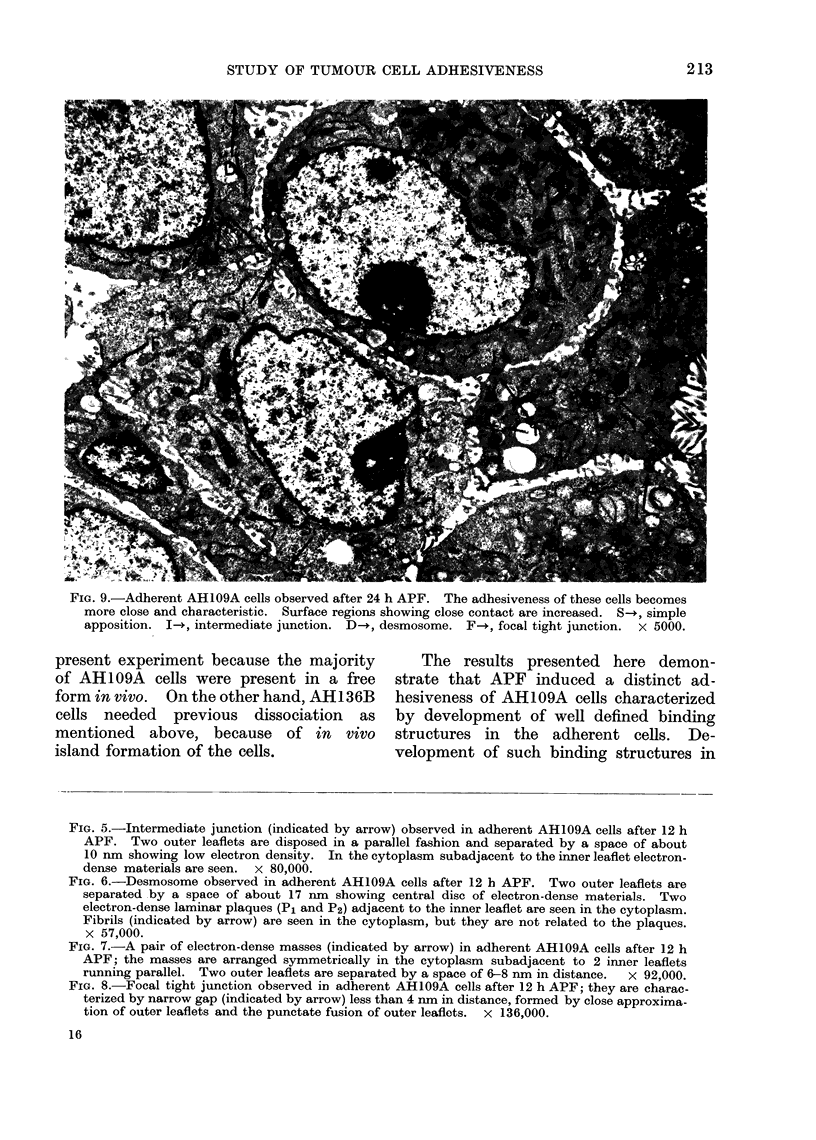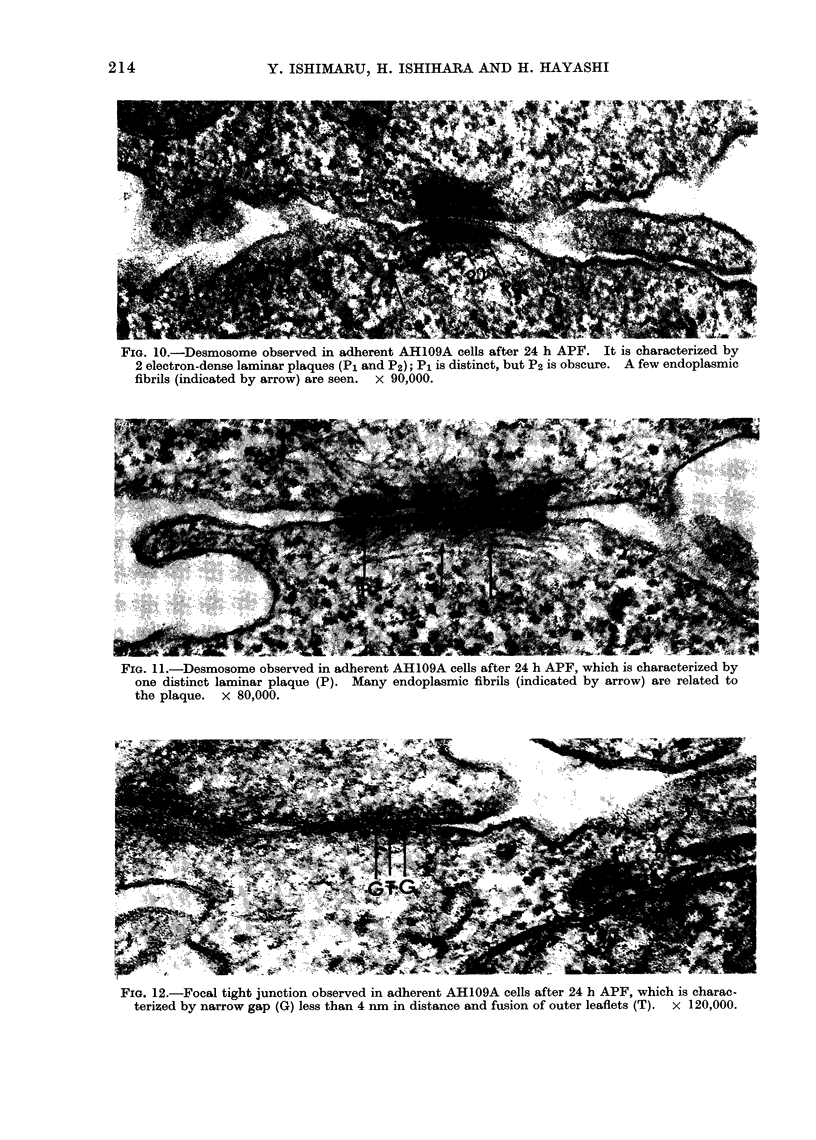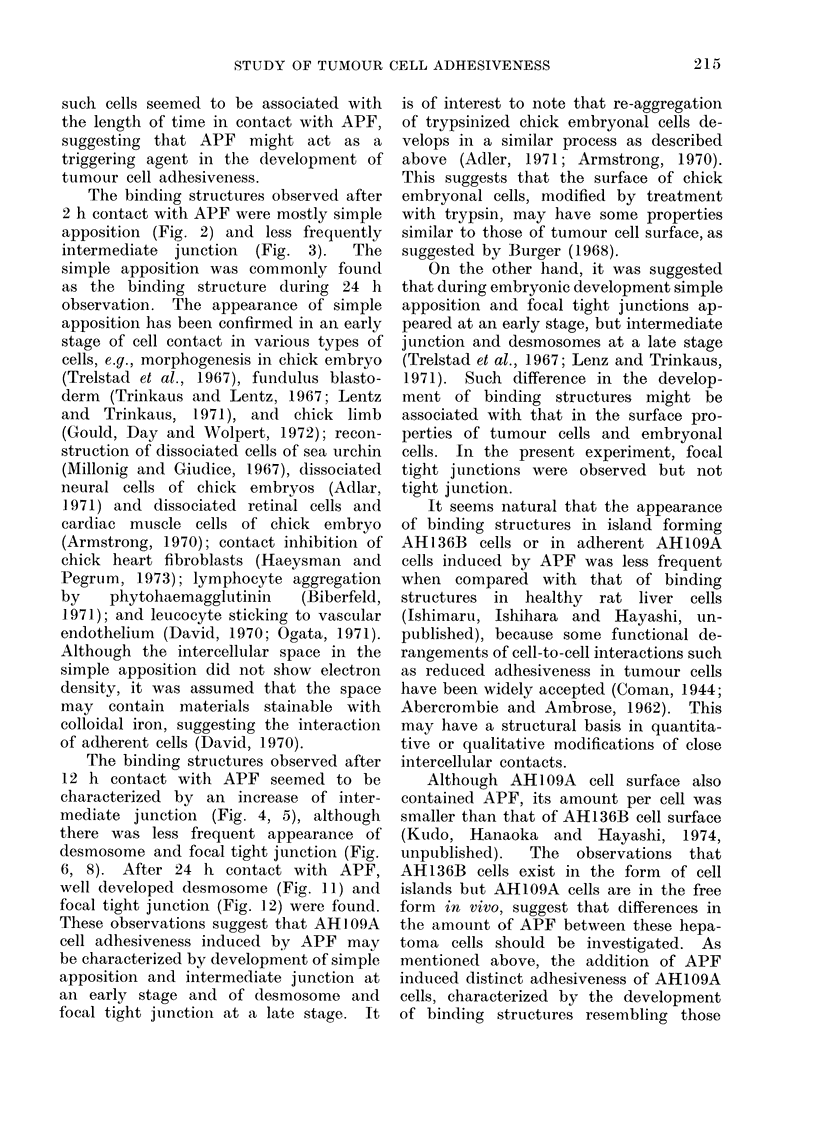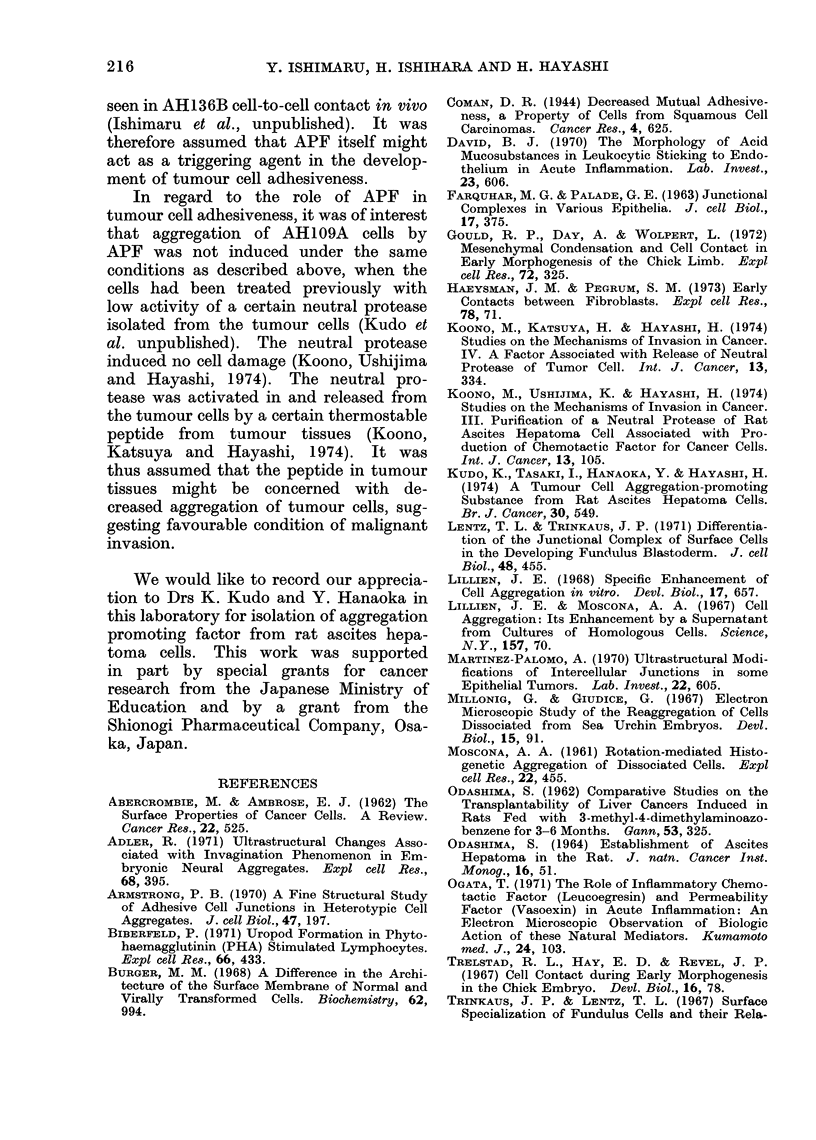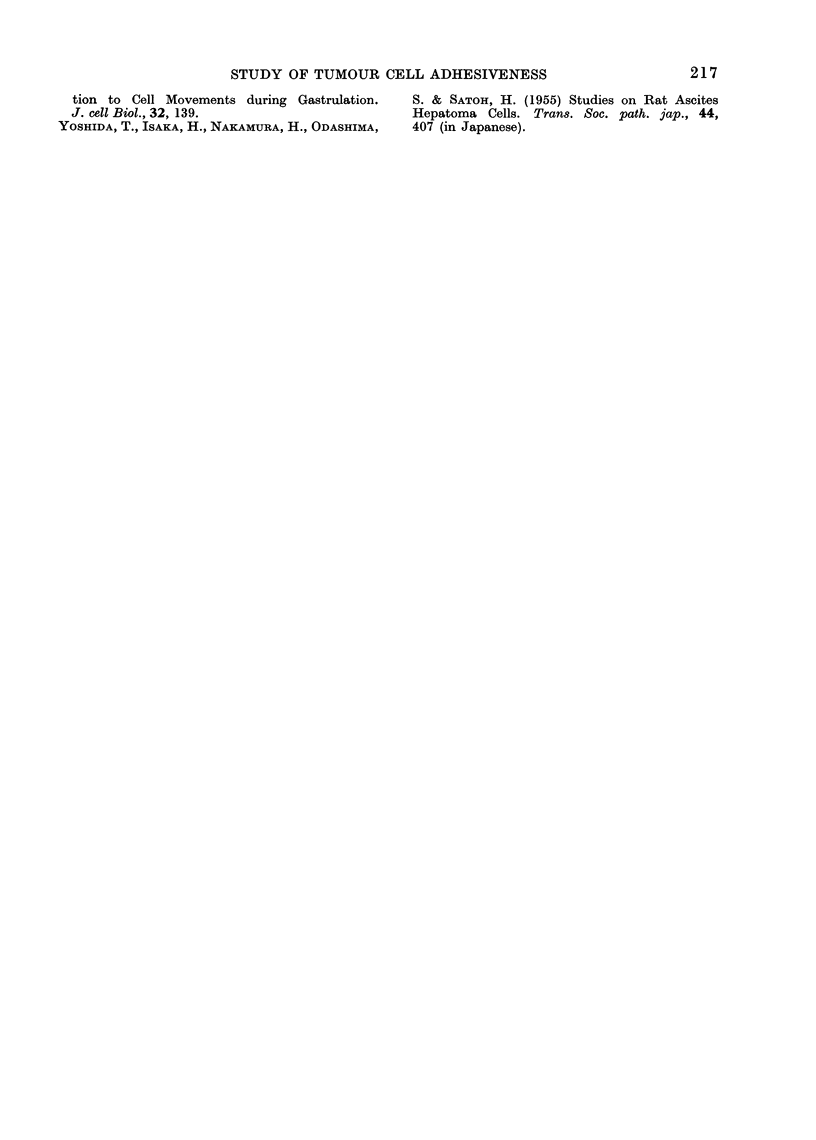# An electron microscopic study of tumour cell adhesiveness induced by aggregation promoting factor from rat ascites hepatoma cells.

**DOI:** 10.1038/bjc.1975.27

**Published:** 1975-02

**Authors:** Y. Ishimaru, H. Ishihara, H. Hayashi

## Abstract

**Images:**


*Br. J. Cancer* (1975) **31**, 207

### AN ELECTRON MICROSCOPIC STUDY OF TUMOUR CELL ADHESIVENESS INDUCED BY AGGREGATION PROMOTING FACTOR FROM RAT ASCITES HEPATOMA CELLS*

Y. ISHIMARU, H. ISHIHARA AND H. HAYASHI

*From Department of Pathology, Kumamoto University Medical School, Kumamoto 860, Japan*

Received 13 September 1974. Accepted 21 October 1974

**Summary.—A substance capable of inducing tumour cell aggregation, which is supposed to be a glycoprotein showing noncytotoxicity, was separated from rat ascites hepatoma cells and partially purified by chromatography. Adhesiveness of rat ascites hepatoma cells induced by this substance was characterized by gradual development of known binding structures during a period of 24 h after contact with the substance; simple apposition and intermediate junctions developed in the early stage, and desmosomes and focal tight junctions in the later stage. It was assumed that the substance might be involved in the development of such binding structures as a triggering mechanism of tumour cell adhesiveness.**

As PREVIOUSLY described (Kudo *et al.*, 1974), a substance capable of promoting tumour cell aggregation has been separated from the surface of rat ascites hepatoma cells and partially purified by chromatography. The substance was non-cytotoxic and clearly effective for adhesiveness of rat ascites hepatoma cells as well as SV40 transformed cells, but not for normal rat liver cells and red blood cells. It was assumed to be a glycoprotein with a molecular weight of about 72,000. The action of this material was clearly more potent than that of concanavalin A (con A). Its effect was not influenced by con A inhibitor such as alpha methyl-D-glucopyranoside, N-acetyl-D-glucosamine and D-glucose.

As is well known, the mechanisms which control cell adhesiveness have been suggested as being intimately related to the surface properties of the cells, and specific sugar containing macromolecular constituents of the cell surface have been suggested to mediate cell adhesiveness (Lilien and Moscona, 1967; Lilien, 1968). The mechanisms of tumour cell adhesiveness appear to be important for explaining malignant invasion. The purpose of the present communication is to describe the light and electron microscopic process of rat ascites hepatoma cell adhesiveness induced by this substance.

### MATERIALS AND METHODS

*Rat ascites hepatomata.*—Rat ascites hepatoma AH136B (Odashima, 1962) and AH109A (Odashima, 1964) have been maintained in our laboratory by routine passage of $1 \times 10^6$ AH136B cells or $2 \times 10^6$ AH109A cells injected i.p. into 80–100 g male rats of Donryu strain. Most (about 98%) of the AH136B cells were found to form cell islands of varying size *in vivo*. On the other hand, most (about 98%) of the AH109A cells were found to be free *in vivo*.

*Preparation of cell suspension.*—AH109A cell suspension was prepared as follows: The ascitic fluid (20 ml) was withdrawn by i.p. puncture 7 days after inoculation of AH109A cells and diluted 1 : 5 with 0·45% NaCl solution. The cell suspension was kept at room temperature for 60 min to allow red blood cells to separate, and tumour cells were sedimented by centrifugation at

* This is No. 2 of the studies on tumour cell aggregation promoting factor.

25 *g* for 10 min. After washings with 0·45% NaCl, the cells were finally suspended at a concentration of $2 \times 10^6$ cells/ml in Earle's MEM containing 20% normal rat serum.

AH136B cell suspension was prepared according to the method previously described (Kudo *et al.*, 1974). The ascitic fluid (20 ml), collected by i.p. puncture 10 days after inoculation of AH136B cells, was diluted 1 : 5 with 0·45% NaCl. After separation of red blood cells by keeping for 60 min at room temperature, tumour cell islands were sedimented by centrifugation and washed with 0·45% NaCl. The cell islands, suspended in Hanks' balanced salt solution (free of calcium and magnesium) containing 0·1 mmol EDTA, were dissociated mechanically by gentle pipetting. Finally, the cell suspension containing $2 \times 10^5$ cells/ml was prepared in Hanks' balanced salt solution; most of the cells in the suspension were found to be free and the remaining cells (about 10%) found in the form of small island composed of only 2–5 cells.

*Isolation of aggregation promoting factor (APF).*—This was performed essentially by the method previously described (Kudo *et al.*, 1974). APF was released from $15 \times 10^8$ AH136B cells, suspended in Hanks' balanced salt solution (free of calcium and magnesium) in the cold, by treatment with 50 gentle " pipettings " and partially purified by chromatography using DEAE–Sephadex and by gel filtration using Bio-gel. The substance was then made up in Earle's MEM at a concentration of 0·15, measured as absorbance at 280 nm/ml. Before use, APF solution was filtered through Millipore filters (pore size 0·3 $\mu$m).

In vitro *induction of tumour cell aggregation.*—This was carried out essentially by a modification (Kudo *et al.*, 1974) of the method of Moscona (1961). Equal volumes (1·5 ml) of APF solution and tumour cell suspension were mixed in a Falcon tube (1·5 × 9·5 cm) and incubated at 37°C in a roller tube culture apparatus, model Te-Her (Hirasawa Co., Tokyo, Japan) run at one rotation/8 min. At intervals of 2, 12 and 24 h after addition of APF, cell aggregates formed were removed by a pipette from the Falcon tubes for light and electron microscopic examination.

*Electron microscopy.*—Immediately after removal from Falcon tubes, the aggregated cells were placed in cold 4% glutaraldehyde in 0·1 mol S-collidin buffer (pH 7·3–7·4) for 45 min. The cells were rinsed with cold 0·1 mol S-collidin buffer and then fixed in cold 2% osmium tetroxide in 0·1 mol S-collidin buffer for 45 min. The cells fixed were stained with 2% uranyl-acetate in distilled water to enhance membrane and fibrillar structures for 60 min at room temperature. The cells were dehydrated with graded alcohol and embedded in Epon 812 in the usual way. Thin sections cut with a Porter-Blum MT-1 microtome (Ivan Sorvall Inc., Norwalk, Conn., U.S.A.) were stained with lead acetate, mounted on 150 mesh grids coated with collodion film and examined in a Hitachi HU-11A electron microscope (Hitachi Ltd, Tokyo, Japan). Measurements were made with a magnifying measuring eyepiece on prints of known enlargement. Thick sections were also prepared for light microscopy and stained with haematoxylin and eosin or trypan blue.

### RESULTS

### I. *Light microscopic observation of tumour cell aggregation*

Equal volumes (1·5 ml) of APF solution and tumour cell suspension were mixed and incubated at 37°C. Induction of macroscopic aggregation of AH109A cells at concentration of $2 \times 10^6$ cells/ml became visible at an early stage, after 10 min of incubation. After further incubation, the cell aggregates became larger, fused with each other and after 30 min incubation time sedimented as a mass on the bottom of Falcon tubes. The cell aggregates removed were fixed and stained with aceto-gentian violet solution prepared by the method of Yoshida *et al.* (1955). The aggregated cells showed a tendency to arrange in a concentric pattern (Fig. 1a). On the other hand, no induction of cell aggregation was revealed in the absence of APF (Fig. 1b). Similar results were obtained with AH136B cells at a concentration of $2 \times 10^5$ cells/ml. These observations indicated that such aggregated AH109A cells were useful for electron microscopic examination and that these cells were,

FIG. 1a.—Light microphotograph of a part of aggregated AH109A cells after 30 min incubation with APF. Stained with aceto-gentian violet. × 120.

FIG. 1b.—Light microphotograph of AH109A cells in a free form in the absence of APF after 30 min incubation. × 120.

when assayed at concentration of $2 \times 10^6$ cells/ml, more convenient than the AH136B cells forming cell islands, because most of the AH109A cells are originally free *in vivo.*

II. *Electron microscopic observation of AH109A cell adhesiveness*

(a) *Cell adhesiveness at 2 h after contact with APF.*—After 2 h incubation, the most common sort of cell contact observed was simple apposition of plasma membranes as described by Farquhar and Palade (1963). Apposed plasma membranes were separated by a space of 10–30 nm showing no electron density (Fig. 2). The structure consisted of 2 outer leaflets disposed in a parallel fashion, showing focal membrane undulation of varying degree. The intermediate junction, although less frequent, consisted of 2 outer leaflets disposed in a parallel fashion and separated by intercellular space less than 20 nm exhibiting low electron density (Fig. 3), which resembles that described by Farquhar and Palade (1963). In the cytoplasm subadjacent to the inner leaflets, moderate electron density was revealed (Fig. 3). At this stage of cell adhesiveness, no desmosome- or tight junction-like structures were observable.

(b) *Cell adhesiveness at 12 h after contact with APF.*—After 12 h incubation, AH109A cell adhesiveness became closer and more distinct. In addition to simple apposition, the cell contact seemed to be characterized by an increase of intermediate junctions (Figs. 4, 5); the frequency of simple apposition and intermediate junction observed at this stage seemed to be in the ratio of 10 : 7.

2

3

4

Desmosome- and tight junction-like structures, resembling those described by Farquhar and Palade (1963), Trelstad, Hay and Revel (1967) and Martinez-Palomo (1970), were further found only in the limited surface region of close cell contact. The desmosome-like structures consisted of 2 outer leaflets running in a parallel fashion and separated by an intercellular space of about 17 nm containing a central disc of electron-dense materials (Fig. 6). In the cytoplasm subadjacent to each inner leaflet, electron-dense laminar plaques running parallel to the inner leaflets were observed (Fig. 6). A few fibrils were frequently found in the cytoplasm but they seemed to be unrelated to the laminar plaques described above. Such structures seemed to be desmosomes in process of development. Occasionally, electron-dense masses were revealed symmetrically in the cytoplasm subadjacent to the inner leaflets, which resemble a pair of button-like developments described by Lentz and Trinkaus (1971); the intercellular space was observed to be 6–8 nm (Fig. 7).

A focal tight junction, as described by Trelstad *et al.* (1967), was observed less frequently at this stage and it was characterized by a narrow gap less than 4 nm in distance which was formed by close approximation of outer leaflets and punctate fusion of outer leaflets (Fig. 8).

(c) *Cell adhesiveness at 24 h after contact with APF.*—After 24 h incubation, AH109A cell adhesiveness became closer and characteristic; the cell surface regions showing close contact were clearly increased (Fig. 9). In addition to simple apposition and intermediate junctions, the cell contact was characterized by an increase of desmosomes and focal tight junctions. Desmosome-like structures observed at this stage seemed to be divided into 3 types: (1) desmosomes were characterized by 2 electron-dense laminar plaques which were not accompanied by endoplasmic fibrils, like those observed after 12 h in contact with APF; (2) desmosomes characterized by one distinct laminar plaque and one obscure laminar plaque accompanied by a few endoplasmic fibrils (Fig. 10); and (3) well defined desmosomes characterized by one distinct laminar plaque accompanied by prominent endoplasmic fibrils (Fig. 11). In general, the outer leaflets seemed to exhibit electron density higher than that of the inner leaflets. Well defined focal tight junctions were occasionally revealed in the limited surface regions of close cell contact (Fig. 12). The frequency of simple apposition, intermediate junction, desmosome and focal tight junctions observed at this stage seemed to be in the ratio of 10 : 7 : 2·5 : 0·3 in that order when counted for 100 cells.

### DISCUSSION

APF, which was separated from AH136B cells forming cell islands *in vivo*, showed a similar effect for aggregation of AH109A cells existing as free cells *in vivo*, when tested at concentration of $2 \times 10^6$ cells/ml (Fig. 1), as shown with AH136B cells at concentration of $2 \times 10^5$ cells/ml (Kudo *et al.*, 1974). This was clearly convenient for the

FIG. 2.—Simple apposition observed in adherent AH109A cells after 2 h contact with APF. Two plasma membranes are disposed in a parallel fashion and separated by intercellular space of about 20 nm in distance. The specialized junctional structures are not seen in this element. This type of cell adhesiveness is most frequently found. × 40,000.

FIG. 3.—Intermediate junction (indicated by arrow) observed in adherent AH109A cells after 2 h APF. Two plasma membranes are disposed in a parallel fashion and separated by a space of about 10 nm showing low electron density. In the cytoplasm subadjacent to the plasma membranes electron-dense materials are seen. × 43,000.

FIG. 4.—Adherent AH109A cells after 12 h APF. The adhesiveness of these cells becomes more close and distinct. Surface regions showing close contact are increased. S→, simple apposition. I→, intermediate junction. D→, desmosome. × 5000.

5

6

7

8

FIG. 9.—Adherent AH109A cells observed after 24 h APF. The adhesiveness of these cells becomes more close and characteristic. Surface regions showing close contact are increased. S→, simple apposition. I→, intermediate junction. D→, desmosome. F→, focal tight junction. × 5000.

present experiment because the majority of AH109A cells were present in a free form *in vivo*. On the other hand, AH136B cells needed previous dissociation as mentioned above, because of *in vivo* island formation of the cells.

The results presented here demonstrate that APF induced a distinct adhesiveness of AH109A cells characterized by development of well defined binding structures in the adherent cells. Development of such binding structures in

---

FIG. 5.—Intermediate junction (indicated by arrow) observed in adherent AH109A cells after 12 h APF. Two outer leaflets are disposed in a parallel fashion and separated by a space of about 10 nm showing low electron density. In the cytoplasm subadjacent to the inner leaflet electron-dense materials are seen. × 80,000.

FIG. 6.—Desmosome observed in adherent AH109A cells after 12 h APF. Two outer leaflets are separated by a space of about 17 nm showing central disc of electron-dense materials. Two electron-dense laminar plaques ($P_1$ and $P_2$) adjacent to the inner leaflet are seen in the cytoplasm. Fibrils (indicated by arrow) are seen in the cytoplasm, but they are not related to the plaques. × 57,000.

FIG. 7.—A pair of electron-dense masses (indicated by arrow) in adherent AH109A cells after 12 h APF; the masses are arranged symmetrically in the cytoplasm subadjacent to 2 inner leaflets running parallel. Two outer leaflets are separated by a space of 6–8 nm in distance. × 92,000.

FIG. 8.—Focal tight junction observed in adherent AH109A cells after 12 h APF; they are characterized by narrow gap (indicated by arrow) less than 4 nm in distance, formed by close approximation of outer leaflets and the punctate fusion of outer leaflets. × 136,000.

FIG. 10.—Desmosome observed in adherent AH109A cells after 24 h APF. It is characterized by 2 electron-dense laminar plaques ($P_1$ and $P_2$); $P_1$ is distinct, but $P_2$ is obscure. A few endoplasmic fibrils (indicated by arrow) are seen. × 90,000.

FIG. 11.—Desmosome observed in adherent AH109A cells after 24 h APF, which is characterized by one distinct laminar plaque (P). Many endoplasmic fibrils (indicated by arrow) are related to the plaque. × 80,000.

FIG. 12.—Focal tight junction observed in adherent AH109A cells after 24 h APF, which is characterized by narrow gap (G) less than 4 nm in distance and fusion of outer leaflets (T). × 120,000.

such cells seemed to be associated with the length of time in contact with APF, suggesting that APF might act as a triggering agent in the development of tumour cell adhesiveness.

The binding structures observed after 2 h contact with APF were mostly simple apposition (Fig. 2) and less frequently intermediate junction (Fig. 3). The simple apposition was commonly found as the binding structure during 24 h observation. The appearance of simple apposition has been confirmed in an early stage of cell contact in various types of cells, *e.g.*, morphogenesis in chick embryo (Trelstad *et al.*, 1967), fundulus blastoderm (Trinkaus and Lentz, 1967; Lentz and Trinkaus, 1971), and chick limb (Gould, Day and Wolpert, 1972); reconstruction of dissociated cells of sea urchin (Millonig and Giudice, 1967), dissociated neural cells of chick embryos (Adlar, 1971) and dissociated retinal cells and cardiac muscle cells of chick embryo (Armstrong, 1970); contact inhibition of chick heart fibroblasts (Haeysman and Pegrum, 1973); lymphocyte aggregation by phytohaemagglutinin (Biberfeld, 1971); and leucocyte sticking to vascular endothelium (David, 1970; Ogata, 1971). Although the intercellular space in the simple apposition did not show electron density, it was assumed that the space may contain materials stainable with colloidal iron, suggesting the interaction of adherent cells (David, 1970).

The binding structures observed after 12 h contact with APF seemed to be characterized by an increase of intermediate junction (Fig. 4, 5), although there was less frequent appearance of desmosome and focal tight junction (Fig. 6, 8). After 24 h contact with APF, well developed desmosome (Fig. 11) and focal tight junction (Fig. 12) were found. These observations suggest that AH109A cell adhesiveness induced by APF may be characterized by development of simple apposition and intermediate junction at an early stage and of desmosome and focal tight junction at a late stage. It is of interest to note that re-aggregation of trypsinized chick embryonal cells develops in a similar process as described above (Adler, 1971; Armstrong, 1970). This suggests that the surface of chick embryonal cells, modified by treatment with trypsin, may have some properties similar to those of tumour cell surface, as suggested by Burger (1968).

On the other hand, it was suggested that during embryonic development simple apposition and focal tight junctions appeared at an early stage, but intermediate junction and desmosomes at a late stage (Trelstad *et al.*, 1967; Lenz and Trinkaus, 1971). Such difference in the development of binding structures might be associated with that in the surface properties of tumour cells and embryonal cells. In the present experiment, focal tight junctions were observed but not tight junction.

It seems natural that the appearance of binding structures in island forming AH136B cells or in adherent AH109A cells induced by APF was less frequent when compared with that of binding structures in healthy rat liver cells (Ishimaru, Ishihara and Hayashi, unpublished), because some functional derangements of cell-to-cell interactions such as reduced adhesiveness in tumour cells have been widely accepted (Coman, 1944; Abercrombie and Ambrose, 1962). This may have a structural basis in quantitative or qualitative modifications of close intercellular contacts.

Although AH109A cell surface also contained APF, its amount per cell was smaller than that of AH136B cell surface (Kudo, Hanaoka and Hayashi, 1974, unpublished). The observations that AH136B cells exist in the form of cell islands but AH109A cells are in the free form *in vivo*, suggest that differences in the amount of APF between these hepatoma cells should be investigated. As mentioned above, the addition of APF induced distinct adhesiveness of AH109A cells, characterized by the development of binding structures resembling those seen in AH136B cell-to-cell contact *in vivo* (Ishimaru *et al.*, unpublished). It was therefore assumed that APF itself might act as a triggering agent in the development of tumour cell adhesiveness.

In regard to the role of APF in tumour cell adhesiveness, it was of interest that aggregation of AH109A cells by APF was not induced under the same conditions as described above, when the cells had been treated previously with low activity of a certain neutral protease isolated from the tumour cells (Kudo *et al.* unpublished). The neutral protease induced no cell damage (Koono, Ushijima and Hayashi, 1974). The neutral protease was activated in and released from the tumour cells by a certain thermostable peptide from tumour tissues (Koono, Katsuya and Hayashi, 1974). It was thus assumed that the peptide in tumour tissues might be concerned with decreased aggregation of tumour cells, suggesting favourable condition of malignant invasion.

We would like to record our appreciation to Drs K. Kudo and Y. Hanaoka in this laboratory for isolation of aggregation promoting factor from rat ascites hepatoma cells. This work was supported in part by special grants for cancer research from the Japanese Ministry of Education and by a grant from the Shionogi Pharmaceutical Company, Osaka, Japan.

### REFERENCES

ABERCROMBIE, M. & AMBROSE, E. J. (1962) The Surface Properties of Cancer Cells. A Review. *Cancer Res.*, **22**, 525.

ADLER, R. (1971) Ultrastructural Changes Associated with Invagination Phenomenon in Embryonic Neural Aggregates. *Expl cell Res.*, **68**, 395.

ARMSTRONG, P. B. (1970) A Fine Structural Study of Adhesive Cell Junctions in Heterotypic Cell Aggregates. *J. cell Biol.*, **47**, 197.

BIBERFELD, P. (1971) Uropod Formation in Phytohaemagglutinin (PHA) Stimulated Lymphocytes. *Expl cell Res.*, **66**, 433.

BURGER, M. M. (1968) A Difference in the Architecture of the Surface Membrane of Normal and Virally Transformed Cells. *Biochemistry*, **62**, 994.

COMAN, D. R. (1944) Decreased Mutual Adhesiveness, a Property of Cells from Squamous Cell Carcinomas. *Cancer Res.*, **4**, 625.

DAVID, B. J. (1970) The Morphology of Acid Mucosubstances in Leukocytic Sticking to Endothelium in Acute Inflammation. *Lab. Invest.*, **23**, 606.

FARQUHAR, M. G. & PALADE, G. E. (1963) Junctional Complexes in Various Epithelia. *J. cell Biol.*, **17**, 375.

GOULD, R. P., DAY, A. & WOLPERT, L. (1972) Mesenchymal Condensation and Cell Contact in Early Morphogenesis of the Chick Limb. *Expl cell Res.*, **72**, 325.

HAEYSMAN, J. M. & PEGRUM, S. M. (1973) Early Contacts between Fibroblasts. *Expl cell Res.*, **78**, 71.

KOONO, M., KATSUYA, H. & HAYASHI, H. (1974) Studies on the Mechanisms of Invasion in Cancer. IV. A Factor Associated with Release of Neutral Protease of Tumor Cell. *Int. J. Cancer*, **13**, 334.

KOONO, M., USHIJIMA, K. & HAYASHI, H. (1974) Studies on the Mechanisms of Invasion in Cancer. III. Purification of a Neutral Protease of Rat Ascites Hepatoma Cell Associated with Production of Chemotactic Factor for Cancer Cells. *Int. J. Cancer*, **13**, 105.

KUDO, K., TASAKI, I., HANAOKA, Y. & HAYASHI, H. (1974) A Tumour Cell Aggregation-promoting Substance from Rat Ascites Hepatoma Cells. *Br. J. Cancer*, **30**, 549.

LENTZ, T. L. & TRINKAUS, J. P. (1971) Differentiation of the Junctional Complex of Surface Cells in the Developing Fundulus Blastoderm. *J. cell Biol.*, **48**, 455.

LILLIEN, J. E. (1968) Specific Enhancement of Cell Aggregation *in vitro*. *Devl. Biol.*, **17**, 657.

LILLIEN, J. E. & MOSCONA, A. A. (1967) Cell Aggregation: Its Enhancement by a Supernatant from Cultures of Homologous Cells. *Science, N.Y.*, **157**, 70.

MARTINEZ-PALOMO, A. (1970) Ultrastructural Modifications of Intercellular Junctions in some Epithelial Tumors. *Lab. Invest.*, **22**, 605.

MILLONIG, G. & GIUDICE, G. (1967) Electron Microscopic Study of the Reaggregation of Cells Dissociated from Sea Urchin Embryos. *Devl. Biol.*, **15**, 91.

MOSCONA, A. A. (1961) Rotation-mediated Histogenetic Aggregation of Dissociated Cells. *Expl cell Res.*, **22**, 455.

ODASHIMA, S. (1962) Comparative Studies on the Transplantability of Liver Cancers Induced in Rats Fed with 3-methyl-4-dimethylaminoazobenzene for 3–6 Months. *Gann*, **53**, 325.

ODASHIMA, S. (1964) Establishment of Ascites Hepatoma in the Rat. *J. natn. Cancer Inst. Monog.*, **16**, 51.

OGATA, T. (1971) The Role of Inflammatory Chemotactic Factor (Leucoegresin) and Permeability Factor (Vasoexin) in Acute Inflammation: An Electron Microscopic Observation of Biologic Action of these Natural Mediators. *Kumamoto med. J.*, **24**, 103.

TRELSTAD, R. L., HAY, E. D. & REVEL, J. P. (1967) Cell Contact during Early Morphogenesis in the Chick Embryo. *Devl. Biol.*, **16**, 78.

TRINKAUS, J. P. & LENTZ, T. L. (1967) Surface Specialization of Fundulus Cells and their Relation to Cell Movements during Gastrulation. *J. cell Biol.*, **32**, 139.

YOSHIDA, T., ISAKA, H., NAKAMURA, H., ODASHIMA, S. & SATOH, H. (1955) Studies on Rat Ascites Hepatoma Cells. *Trans. Soc. path. jap.*, **44**, 407 (in Japanese).